# Author Correction: Reconstitution of contractile actomyosin rings in vesicles

**DOI:** 10.1038/s41467-022-29063-4

**Published:** 2022-03-10

**Authors:** Thomas Litschel, Charlotte F. Kelley, Danielle Holz, Maral Adeli Koudehi, Sven K. Vogel, Laura Burbaum, Naoko Mizuno, Dimitrios Vavylonis, Petra Schwille

**Affiliations:** 1 Department of Cellular and Molecular Biophysics, Max Planck Institute of Biochemistry, Martinsried, Germany; 2 Department of Structural Cell Biology, Max Planck Institute of Biochemistry, Martinsried, Germany; 3 Department of Physics, Lehigh University, Bethlehem, PA USA

**Keywords:** Membrane biophysics, Membrane structure and assembly, Actin

Correction to: *Nature Communications* 10.1038/s41467-021-22422-7, published online 15 April 2021.

In this article, the funding from ‘the European Union’s Horizon 2020 research and innovation program under the Marie Sklodowska-Curie grant agreement No. 794162’ to C.K. was omitted. The original article has been corrected.

