# Supplementary material for: Reconstitution of contractile actomyosin rings in vesicles
Source: Nat Commun. 2021 Apr 15;12:2254. doi: 10.1038/s41467-021-22422-7 (PMC8050101; doi:10.1038/s41467-021-22422-7)
Supplement: Supplementary file 1 — Supplementary Information [file 41467_2021_22422_MOESM1_ESM.pdf]

Supplementary Information for

"Reconstitution of contractile actomyosin rings in vesicles"

Thomas Litschel, Charlotte F. Kelley, Danielle Holz, Maral Adeli Koudehi, Sven K. Vogel, Laura Burbaum, Naoko Mizuno, Dimitrios Vavylonis and Petra Schwille

Supplementary Method

Details regarding theoretical modeling

Simulations are based on the Brownian dynamics model of Adeli-Koudehi et al.[1], as summarized below.

Filament representation

Filaments are represented as series of beads separated by $l_0 = 0.1\ \mu m$, representing 37 actin subunits, connected by springs. Bead motion is governed by forces from the spring and bending forces, rigid confining boundary, crosslinking and stochastic forces. The 3D positions, $\mathbf{r}_i$ of the *i* bead is evolved in time according to:

$$\mathbf{F}_i^{spring} + \mathbf{F}_i^{bend} + \mathbf{F}_i^{stoch} + \mathbf{F}_i^{boundary} + \mathbf{F}_i^{atr} = \zeta_b \frac{d\mathbf{r}_i}{dt}, \tag{1}$$

where ζ_b = 0.108 pN s/µm is an effective drag coefficient, which corresponds to a viscosity 350 times higher than water (lower viscosity values become computationally costly to simulate). The spring, bending and stochastic forces are as follows:

$$\mathbf{F}_i^{spring} = -\frac{k}{2}\sum_{j=1}^{N-1} \frac{\partial\left(\left|\mathbf{r}_{j+1} - \mathbf{r}_j\right| - l_0\right)^2}{\partial \mathbf{r}_i}, \tag{2}$$

$$\mathbf{F}_i^{bend} = \frac{\kappa}{l_0}\sum_{j=2}^{N-1} \frac{\partial\left(\mathbf{t}_j \cdot \mathbf{t}_{j-1}\right)}{\partial \mathbf{r}_i}, \tag{3}$$

$$\langle \mathbf{F}_i^{stoch}(t)\mathbf{F}_i^{stoch}(t')\rangle_{\alpha,\beta} = 2k_B T \zeta_b \delta(t - t')\hat{I}_{\alpha\beta}, \tag{4}$$

where N is the number of beads per filament, $\mathbf{t}_j = \left(\mathbf{r}_{j+1} - \mathbf{r}_j\right)/\left|\mathbf{r}_{j+1} - \mathbf{r}_j\right|$ is the local unit tangent vector, $\kappa = k_B T l_p$ is the flexural rigidity, k_B is Boltzmann's constant, T is temperature, l_p is the persistence length of the filament, *k* = 100 pN/µm is the spring constant between filament beads (stiff enough to prevent significant filament extension or compression) and $\hat{I}_{\alpha\beta}$ is the second-order unit tensor (α and β labeling the x, y or z directions).

Attraction force between actin filaments

Crosslinking between filaments is due to a short-range, isotropic attractive potential between filament beads. If bead *i* is within the distance r_{atr} to bead *j*, then the force on bead *i* due to bead *j* is:

$$\mathbf{F}_i^{atr} = -\frac{k_{atr}}{2}\frac{\partial\left(\left|\mathbf{r}_j - \mathbf{r}_i\right| - r_0\right)^2}{\partial r_i} \quad \left(\left|\mathbf{r}_j - \mathbf{r}_i\right| < r_0\right), \tag{5}$$

where the spring constant is k_{atr} and the crosslink equilibrium length is r_0 = 0.012 µm. The parameters k_{atr} and r_{atr} characterize the effective stiffness and range of interaction between filament beads due to cross-linking. For the simulations in this study we used r_{atr}= 0.06 µm, a value that allows bundle formation without significant filament sliding along the bundle.

Boundary conditions and interactions

Filaments are in a spherical confinement with a repulsive hard wall, which was represented by a constant force of magnitude 1 pN normal to the cell boundary exerted on every bead crossing it. In simulations where filaments were attracted to the boundary, a short-range attraction near the spherical hard wall was implemented by the following short-range surface force to each bead: (6)

$$\mathbf{F}_i^{srf} = -\frac{k_{srf}}{2}\frac{\partial\left(\left|\mathbf{R}_{conf}-\mathbf{r}_i\right|-r_0\right)^2}{\partial\mathbf{r}_i} \qquad \left(\left|\mathbf{R}_{conf}-\mathbf{r}_i\right|<r_{srf}\right),$$

where k_{srf}= 3 pN/µm is the corresponding spring constant and r_{srf} = 0.06 µm is the distance from the confinement surface where beads can feel the force. Here $\mathbf{R}_{conf}$ points to the confining sphere boundary in the direction of $\mathbf{r}_i$.

Simulation of polymerization

We start with a fixed number of filament nuclei which elongate over time by addition of beads at one end of the filament. The elongation rate is proportional to the concentration of the remaining bulk monomers, $C(t)$ and the barbed end polymerization rate, $k_+ = 10\ \mu\mathrm{M/s}$. For a fixed filament concentration $C(t)$ decays according to:

$$C(t) = C_{actin}e^{-k_+F_0t} \tag{7}$$

where F_0 is the initial filament nuclei concentration and C_{actin} is the initial bulk actin monomer concentration. In these simulations, all filaments are the same length and we tuned F_0 to get the desired final filament length, taking into consideration that the higher spontaneous nucleation rate of higher actin concentrations leads to shorter filaments, as in Adeli-Koudehi et al.

Implementation of the simulation

We used a *dt* = 1.5×10^{-4} s for Supplementary Fig. 7a, b and Supplementary Fig. 11a, b. We used a smaller value of *dt* = 0.5×10^{-4} s for Fig. 3b, Supplementary Fig. 7c and Supplementary Fig. 11c that have higher persistence length and require a smaller value for numerical stability. The code of Adeli-Koudehi et al. was modified by adding a list of nearest neighbors to check interactions every timestep. The nearest neighbors list was updated ever 50 *dt* to speed up the runtime.

Classification of ring (R) and ring-like (RL) structures in simulations. In the simulations of ring formation as function of confining diameter for *c* = 2 µM and final filament length *L* = 6 µm (Fig. 3, Supplementary Fig. 11b, c, Supplementary Fig. 12c), we see formations that are close to what could be considered a ring, but have some imperfections such as forking small bundles that stick out of the main ring. Such structures are not as frequently observed in experiments. We anticipate that simulations with longer run-times that also allow filament breakage for filaments with high curvature at intersection points would allow a clean single ring to eventually form. We thus labeled such structures as RL. In the simulations with short filaments for *c* = 2 µM and final filament length *L* = 1.2 µm (Fig. 3, Supplementary Fig. 11a), we also labeled as RL structures with small gaps (less the 20% of the circumference) that could also be filled by

diffusion of bundle segments to the main ring over longer simulation times. Explicit examples are shown in Supplementary Fig. 11.

Details regarding experimental analysis

Image processing and analysis of the Z-stack confocal datasets of actin-labelled vesicles was mostly performed using the software ImageJ/Fiji[2,3], complemented with the plugins Image Stabilizer[4] and Squassh[5] (from the MOSAIC ToolSuite update site). The organization of the actin networks in vesicles under the different experimental conditions was characterized using ImageJ/Fiji in combination with SOAX[6,7]. Specifically, the workflow to derive a skeleton model from the confocal volumes was implemented as a combination of a series of ImageJ scripts, each one performing a processing step on all the images acquired. Some parts of analysis required manual intervention, as below outlined. After making the images conform to its input specifications, the software SOAX was launched and controlled through Fiji to determine a model of the filaments in batch mode.

When characterizing actin bundle morphologies, we analyzed vesicles regardless of their content with exception of clear outliers, such as deformed vesicles and vesicles that did not contain any discernable actin bundles.

Image processing for 3D analysis (Fig. 1d and for Fig. 2b)

The confocal z-stacks were deconvolved using the software Huygens Essential (Scientific Volume Imaging), by means of the Classic Maximum Likelihood Estimation algorithm with a theoretical Point Spread Function. In some stacks a drift between planes of either the actin network within the vesicles or the vesicles themselves was compensated with the Image Stabilizer plugin[4]. All the vesicles in each stack were manually selected and then cropped to generate subvolumes, each containing only one vesicle. The subvolumes were filtered and the filaments segmented from them with the Squassh algorithm as implemented in Fiji[5]. The structures were detected using the following options: background removal with a window of 1 µm size; regularization parameter: 0.075; removal of background intensity with threshold determined by the Triangle method; removal of segmented regions smaller than 1 µm linear size; automatic local intensity estimation; Poisson noise model; soft mask applied to final segmentation. Finally the subvolumes were scaled along the z axis to make the voxels isotropic, as required by SOAX, and a mild 3D Gaussian Blur filter (1 pixel sigma) was applied to reduce any artefact from the scaling procedure.

Generation of 3D actin filament network models (Fig. 1d and for Fig. 2b)

A model for the filament network in each vesicle was determined using the software SOAX[7] on the subvolumes of the segmented filaments. The software implements a Stretching Open Active Contours method to compute a centerline (called 'snake' in the software) from each filament, which can be used for quantitative analysis. The batch processing procedure to extract the

centerlines was launched from Fiji, after setting the parameters that controls the algorithm (see SOAX documentation for their definitions) as follows: Intensity Scaling: 0; Gaussian std: 1.2 pixels; Ridge Threshold: 0.007; Minimum Foreground: 1 sigma level; Maximum Foreground: maximum bit depth of the data; Snake Point Spacing: 2 pixel; Init z: True; Minimum Snake Length: 7 µm; Maximum Iterations: 10000; Check Period: 100; Change Threshold: 0.1 pixels; Alpha: 0.03; Beta: 0.5; Gamma: 2; External Factor: 1; Stretch Factor: 0.2; Number of Background Radial Sectors: 8; Radial Near: 0.35 µm; Radial Far: 0.525 µm; Background Z/XY Ratio: 3; Delta: 0.25 µm; Overlap Threshold: 0.313 µm; Grouping Distance Threshold: 0.5 µm; Grouping Delta: 0.813 µm; Minimum Angle for SOAC Linking: 100 degrees; Damp z: True. The batch procedure generated a file for each vesicle, containing the coordinates of the centerlines modeling the actin network, which were then converted to marker model for visualization in UCSF Chimera[8].

Curvature Analysis (Fig. 2b)

The filament models generated by batch processing were then analyzed from the SOAX GUI, using the Filament Curvature Analysis option. This tool divides the models into equally sized segments and then determines a curvature value for each of the segments. The curvature values (in μm^{-1}) from each vesicle were then normalized with respect to the curvature of the respective vesicle membranes, and the values from all the vesicles under the same experimental condition were finally aggregated to generate the bundle curvature distribution. Curvature values less than 1 corresponds to regions where the filaments are flatter than the membrane, and vice versa.

Membrane Proximity Analysis (Fig. 3c, d)

The quantification of the distribution of the actin networks inside the vesicles was performed on the radial intensity profile. The center and the radius of the vesicle was determined semi automatically, starting from two opposite points on the membrane marked manually in Fiji on a maximum intensity projection of the volume (script provided at https://doi.org/10.5281/zenodo.4555840). The radial profile was used to compute the membrane proximity metric, defined as the weighted average of the radii, with the weights given by the intensity at each radius, and the average normalized by the vesicle radius. Values close to one indicate that most of the intensity is found close to the membrane, while a uniform distribution of the intensity within the vesicle gives a membrane proximity value of 0. Smaller values indicate that the intensity is condensed at the vesicle center.

Supplementary Figures

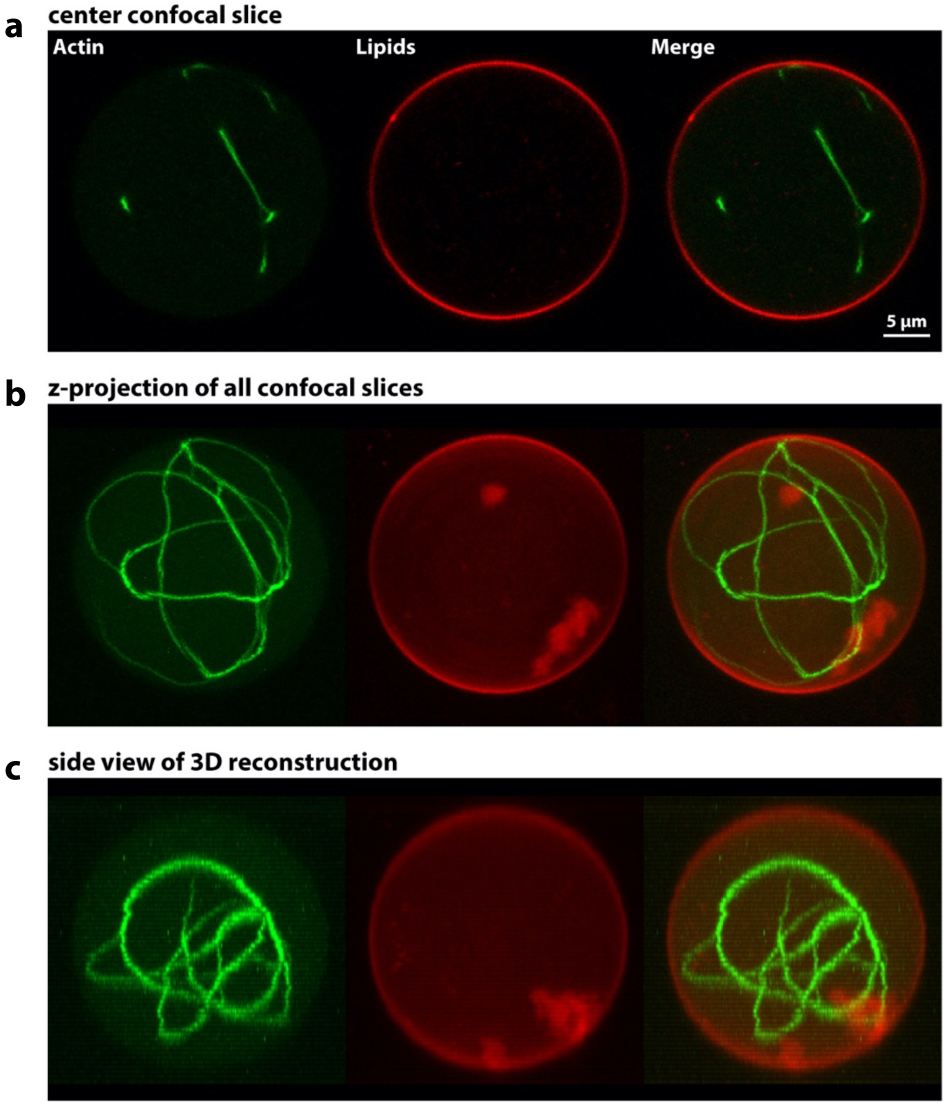

Supplementary Fig. 1: GUV with labelled membrane containing actin bundles bundled by α-actinin. Vesicle is made from POPC with 0.015% DOPE-ATTO655. Images in this paper are taken as z-stacks of confocal slices. **a** Center z-slice of a data set with 62 confocal slices. **b** Projection of all confocal slices. **c** Side-view of a 3D reconstruction of the vesicles.

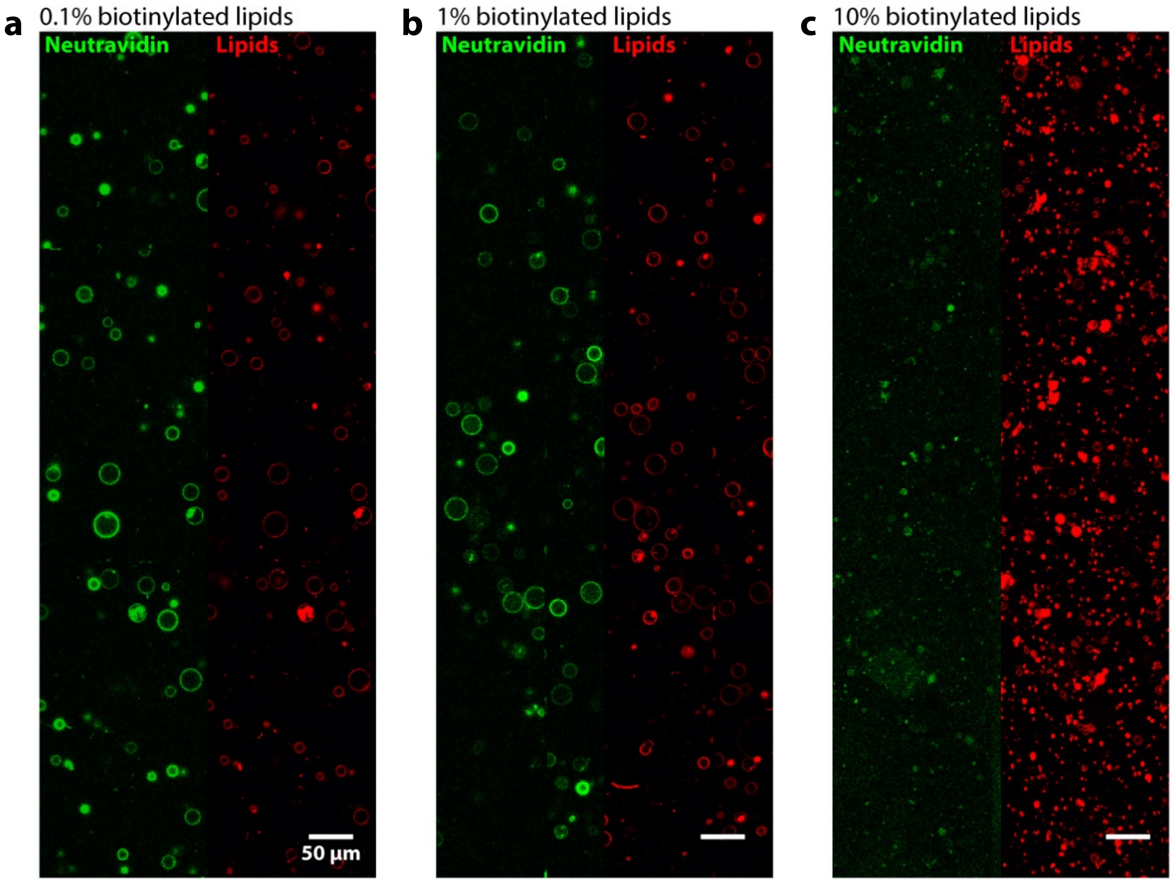

Supplementary Fig. 2: We tested three concentrations of biotinylated lipids (DSPE-PEG(2000) Biotin) in GUVs. **a**, **b** and **c** show fractions of 0.1%, 1% and 10% respectively. The GUVs contain NeutrAvidin™ labeled with OregonGreen™ . OregonGreen is shown in green, lipids (POPC with DOPE-ATTO655) are shown in red. Binding of NeutrAvidin to the membrane seems equally efficient for 0.1% and 1% biotinylated lipids, while the vesicle yield is very low with 10% biotinylated lipids. Most of the lipid signal in (**c**) is not from vesicles, but from lipid aggregates or similar lipid structures.

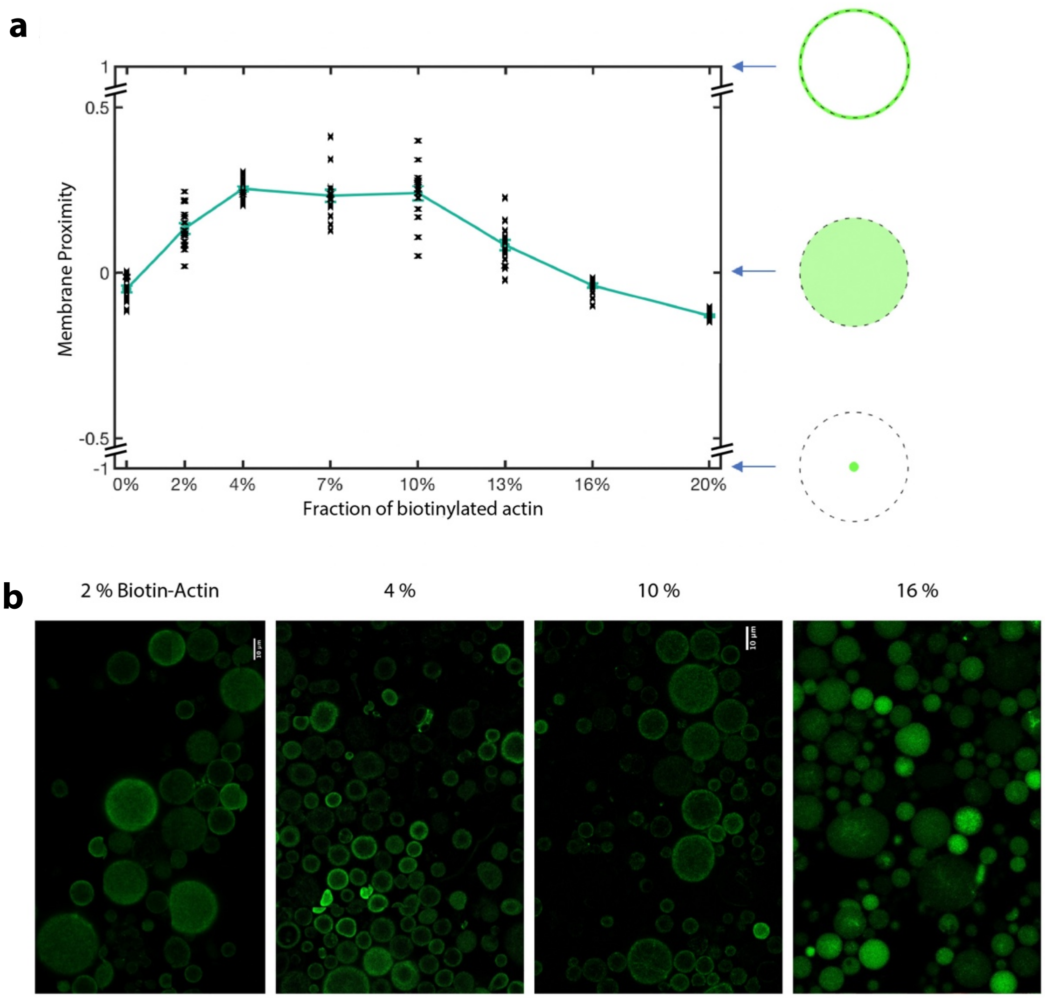

Supplementary Fig. 3: Membrane binding of unbundled actin in dependency of concentration of biotinylated actin. Vesicle membranes contain 1% biotinylated lipid and 0.17 μM Neutravidin. We tested a range of different fractions of biotinylated actin for their effect on binding efficiency in experiments with unbundled actin. Equatorial focal planes of GUVs were analyzed to quantify the average proximity of actin signal to the membrane and plotted on a normalized range from -1 (all signal in the center of the vesicle) to +1 (all signal on the membrane). We found an optimum between 4 % and 10 % biotinylated actin. At fractions of more than 10 %, membrane binding seems to decrease, likely because binding between actin filaments competes with binding to the membrane. n = 15 per condition. Error bars show SEM.

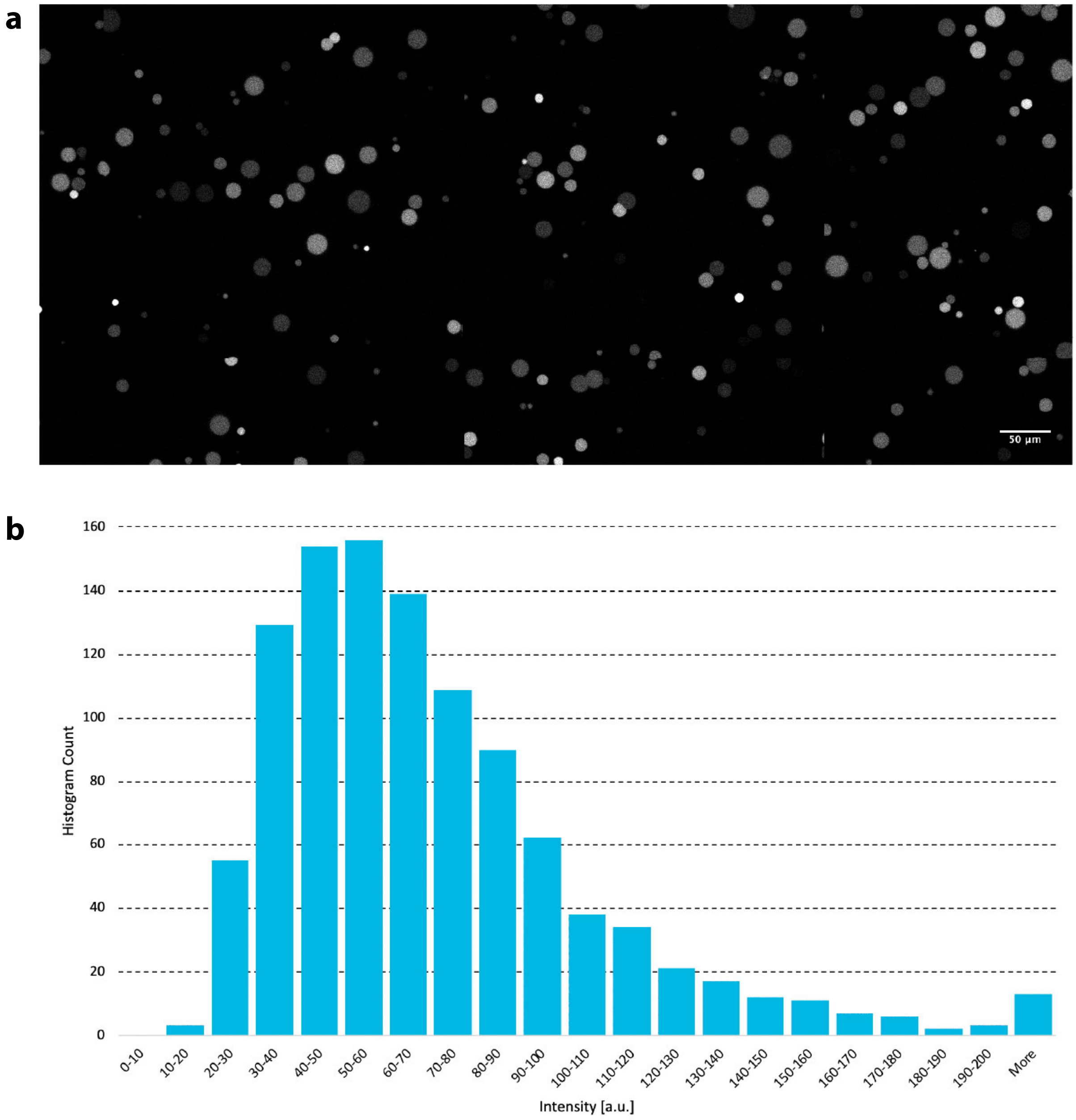

Supplementary Fig. 4: Concentration distribution of encapsulated solubles: Control experiment in which we encapsulate a fluorescent dye (Alexa Fluor 546 NHS-Ester). **a** Field of view section of analyzed vesicles. **b** Fluorescence intensities of 1061 vesicles

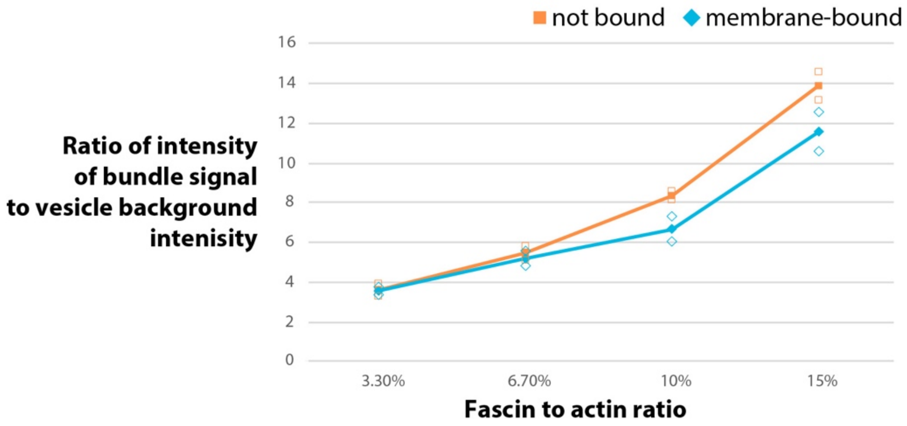

Supplementary Fig. 5: Ratio of bundle signal intensity to background intensity in dependency of fascin to actin ratio in vesicles seen in Fig. 3a. With increasing amounts of fascin, actin bundles get thicker, while the amount of unbundled actin filaments in the vesicle decreases. By plotting the ratio of the fluorescence intensity of the bundles to the intensity of the lumen, we visualize this shift of actin from the bulk phase into bundles. For each condition we analyzed two vesicles. We did 4 measurements per vesicles by plotting the spatial intensity along 4 straight lines (0°,45°, 90°, 135°) and calculated the ratio between highest and lowest intensity within the vesicles. Hollow squares: averages for vesicles. Solid squares: averages for conditions.

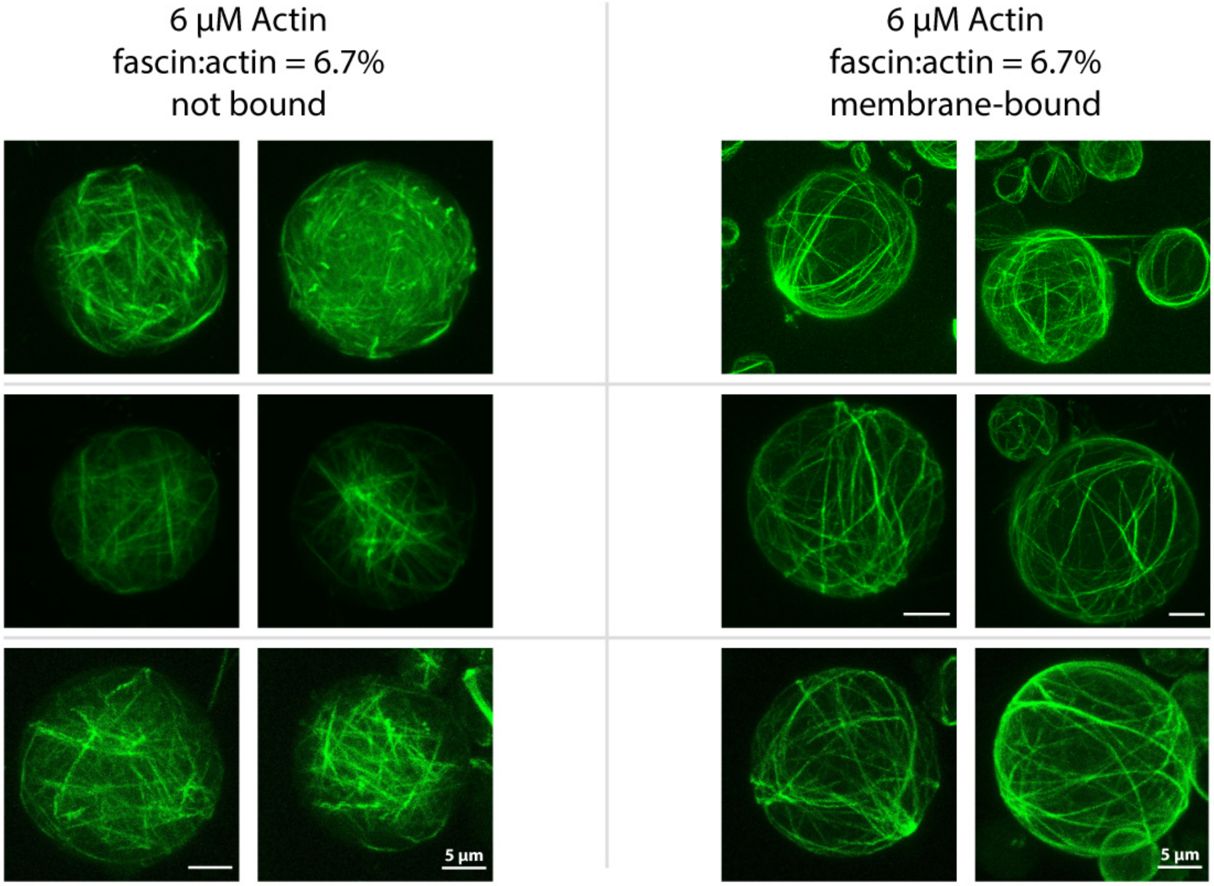

Supplementary Fig. 6: Cytoskeletal vesicles with 6 μM actin and a fascin to actin ratio of 6.7 to 100. For this actin and fascin concentration, in experiments without membrane binding (left), bundles are relatively short and below the threshold for which we see long bundles that wrap around the inner circumference of the vesicle membrane and often connect to other bundles (which is the case for conditions with higher fascin concentrations without membrane-binding, see Fig. 3a). With the addition of membrane binding (right), this threshold seems to be lowered so that bundles can extend to form longer structures. Three separate experimental runs are shown for each condition (each row corresponds to one experimental run).

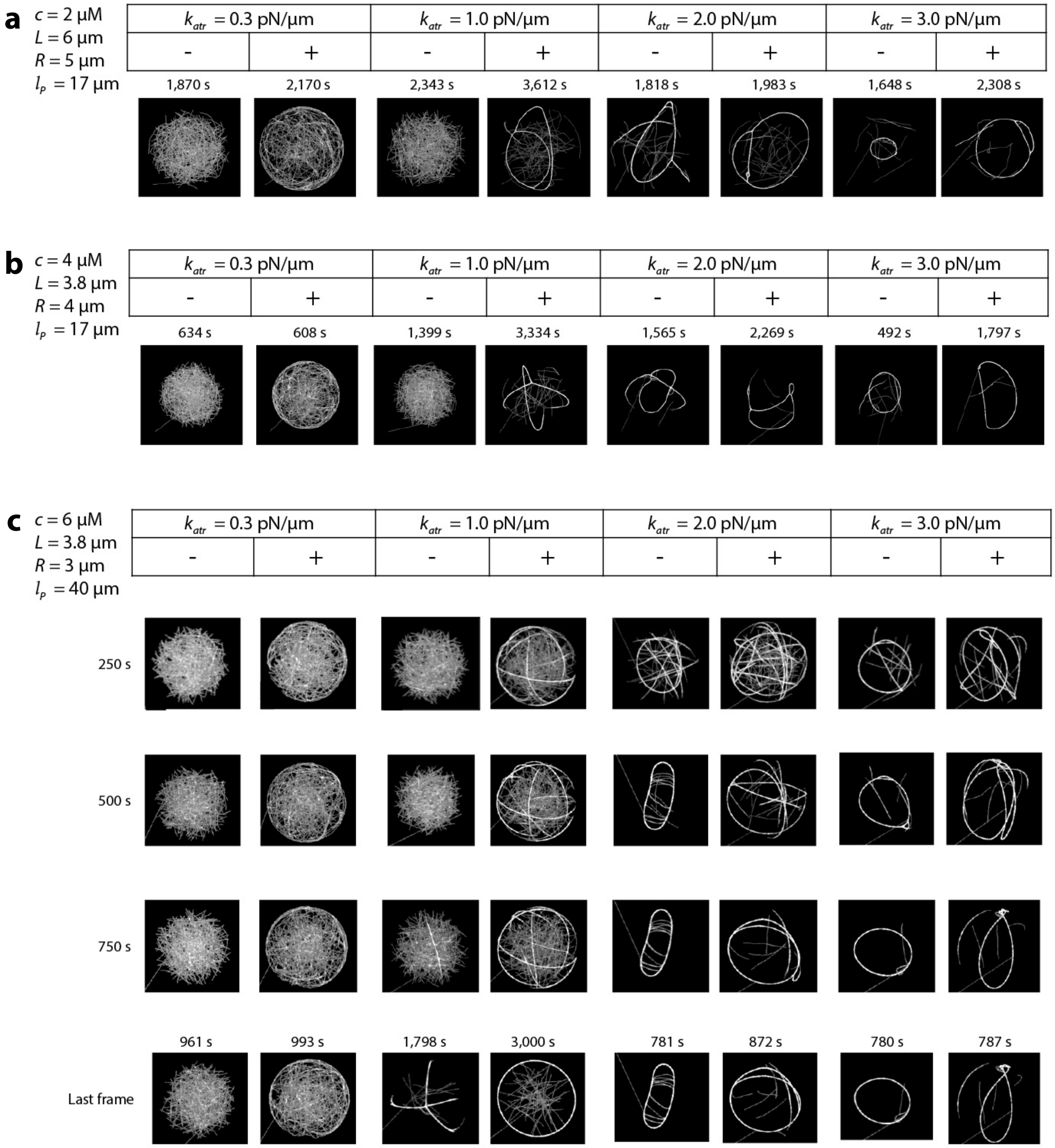

Supplementary Fig. 7: Snapshots (maximum intensity projections) of simulations of confined actin filaments with varying cross-linking interaction strength k_{atr}, with or without attraction to confining boundary, for comparison to experiments of Fig. 3a. All simulations are for final filament lengths L shorter than the confining diameter $2R$, corresponding to experimental measurements. Simulations were run for long enough times such that the bundle/loop configurations reach a stationary shape. Results show a transition from unbundled/weakly bundled to bundled configurations with increasing k_{atr}. Attraction to confining boundary promotes bundling (cases $k_{\mathrm{atr}} = 0.3, 1$ pN/μm) and leads to larger circular structures (cases $k_{\mathrm{atr}} =$

2, 3 pN/μm). The confining radius was decreased with increasing concentration for reasons of computational efficiency. **a** At the lowest actin concentration (c = 2 µM, R = 5 μm, initial number of nuclei 284) single actin rings and ring-like structures are observed at high k_{atr}, similar to bundles of the experiments of Fig. 3a at 2 µM (where fascin promoted bundling at all concentrations). **b** Behavior similar to panel A is seen at intermediate actin concentrations (c = 4 µM, R = 4 μm, initial number of nuclei 498). **c** At the highest actin concentration (c = 6 µM, R = 3 μm, initial number of nuclei 315) and at the higher k_{atr} values, bundle networks at early times resembling the corresponding experimental measurements of Fig. 3a. In panel (**c**) the individual filament persistence length was increased to 40 μm to simulate the effect of stiffening of fascin-actin bundles.

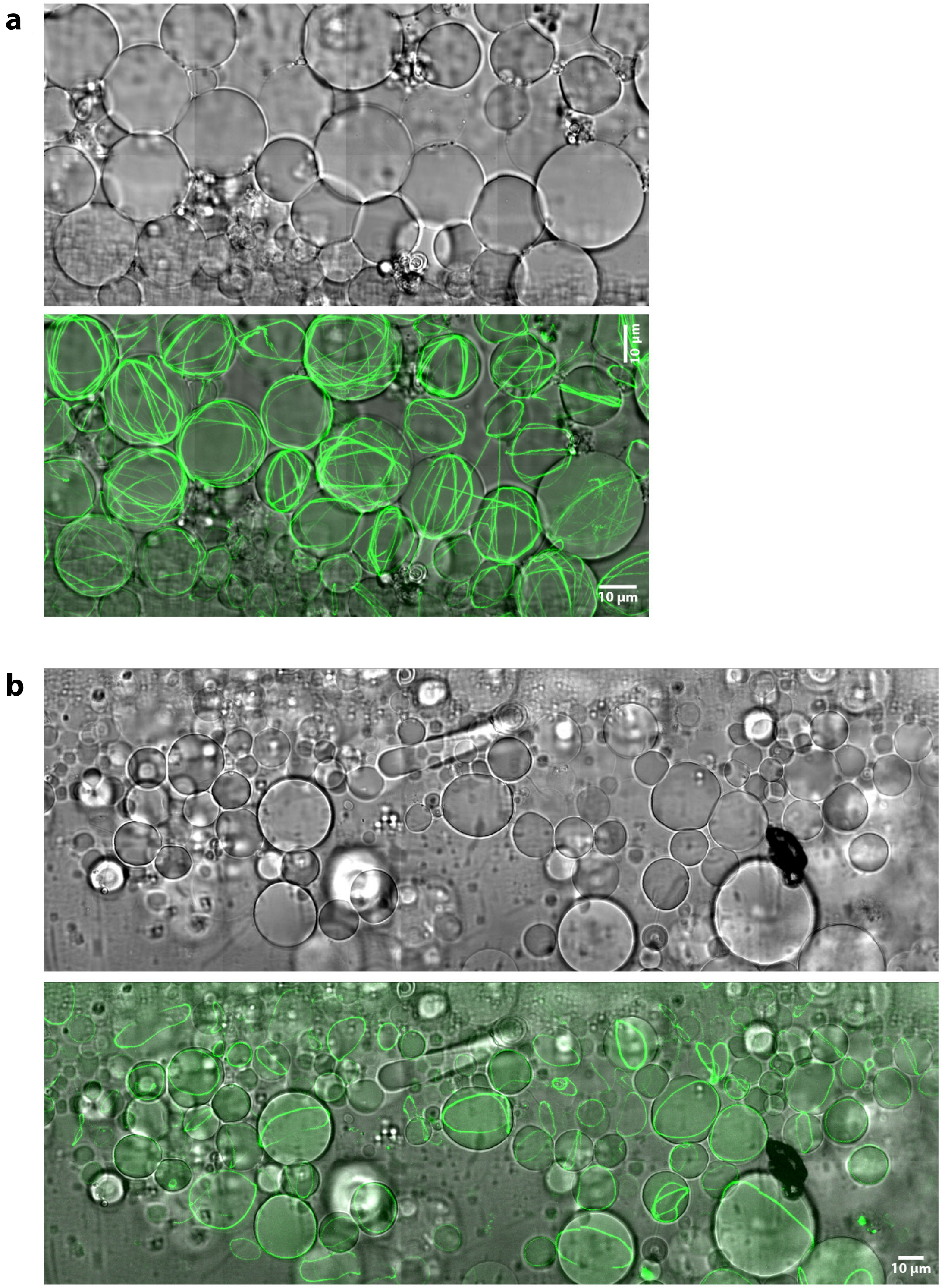

Supplementary Fig. 8: Differential Interference Contrast Images (DIC): **a** Section view from Fig. 1b as DIC image and overlay of fluorescence image (maximum projection of a z-stack of images) on DIC image. **b** Section view from Fig. 4c as DIC image and overlay of fluorescence image (maximum projection of a z-stack of images)

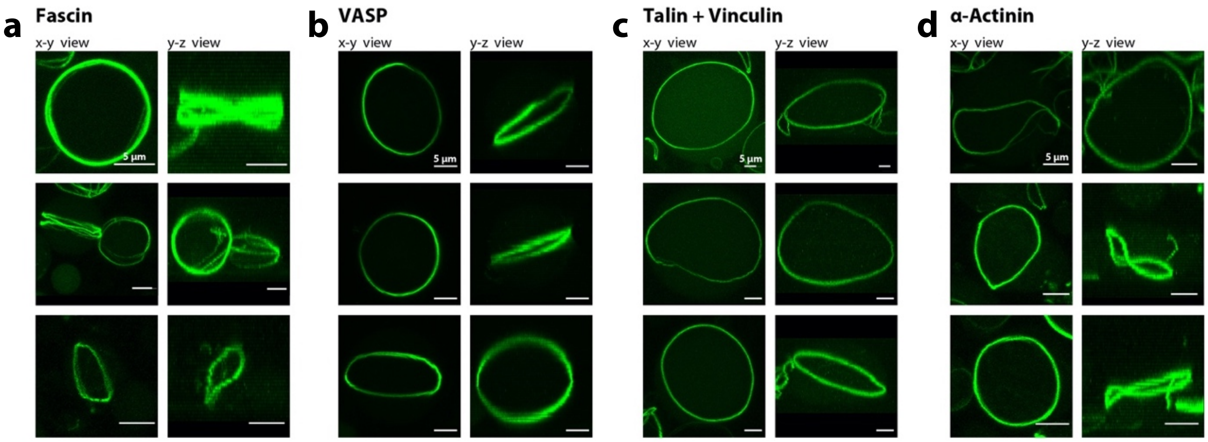

Supplementary Fig. 9: Membrane-bound rings form from actin bundled by fascin (**a**), VASP (**b**), talin with vinculin (**c**) and α-actinin (**d**)). All images are maximum projections of confocal z-stacks. We used 2 μM actin in all cases, but due to differences in bundling activity, different concentrations of bundling protein: 0.3 μM fascin, 0.9 μM VASP, 1 μM α-actinin, 2 μM talin and 2 μM vinculin.

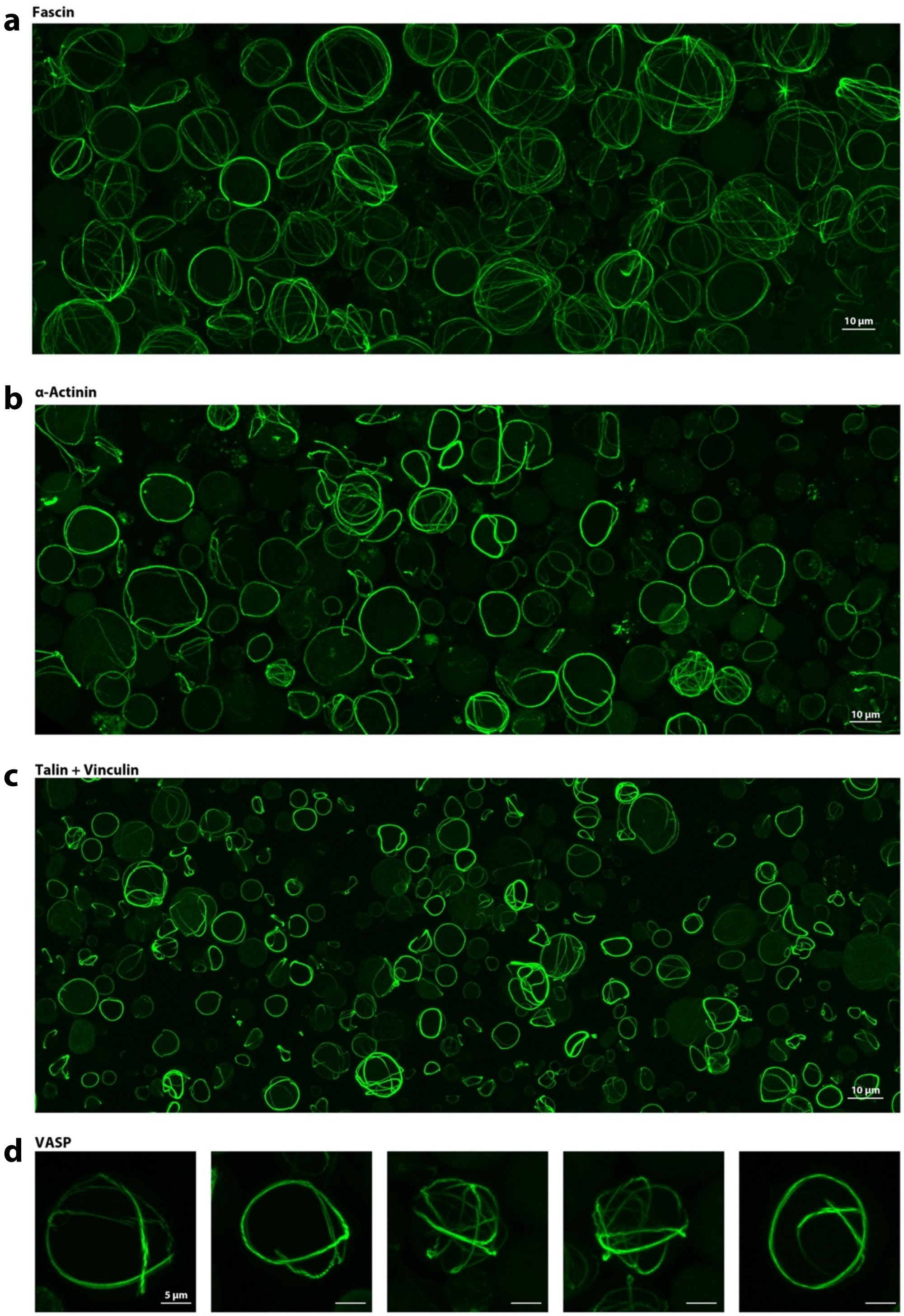

Supplementary Fig. 10: Additional images of vesicles with actin bundled by the four bundlers. Actin is bound to the vesicle membrane by biotin-neutravidin-biotin links. Large area scans of vesicles containing actin bundled by fascin (**a**), α-actinin (**b**) and talin with vinculin (**c**). **d** Images of vesicles with actin bundled by VASP. All images ((**a**)-(**d**)) are maximum projections of confocal z-stacks. We used 2 μM actin in all cases, but due to differences in bundling activity,

different concentrations of bundling protein: 0.3 μM fascin, 0.9 μM VASP, 1 μM α-actinin, 2 μM talin and 2 μM vinculin.

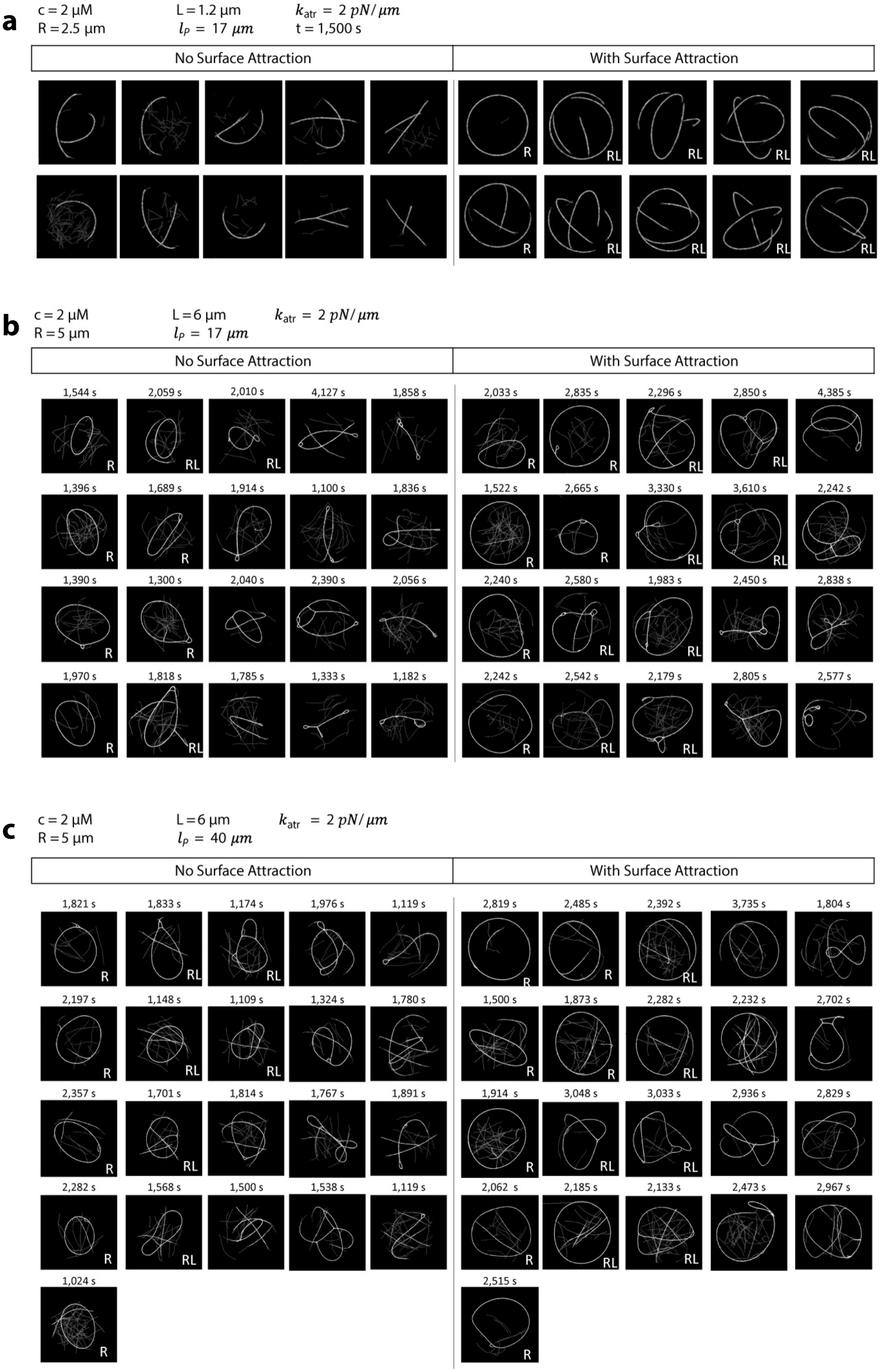
a
c = 2 μM
R = 2.5 μm
L = 1.2 μm
l_P = 17 μm
k_{atr} = 2 pN/μm
t = 1,500 s
No Surface Attraction
With Surface Attraction
b
c = 2 μM
R = 5 μm
L = 6 μm
l_P = 17 μm
k_{atr} = 2 pN/μm
No Surface Attraction
With Surface Attraction
1,544 s 2,059 s 2,010 s 4,127 s 1,858 s 2,033 s 2,835 s 2,296 s 2,850 s 4,385 s
1,396 s 1,689 s 1,914 s 1,100 s 1,836 s 1,522 s 2,665 s 3,330 s 3,610 s 2,242 s
1,390 s 1,300 s 2,040 s 2,390 s 2,056 s 2,240 s 2,580 s 1,983 s 2,450 s 2,838 s
1,970 s 1,818 s 1,785 s 1,333 s 1,182 s 2,242 s 2,542 s 2,179 s 2,805 s 2,577 s
c
c = 2 μM
R = 5 μm
L = 6 μm
l_P = 40 μm
k_{atr} = 2 pN/μm
No Surface Attraction
With Surface Attraction
1,821 s 1,833 s 1,174 s 1,976 s 1,119 s 2,819 s 2,485 s 2,392 s 3,735 s 1,804 s
2,197 s 1,148 s 1,109 s 1,324 s 1,780 s 1,500 s 1,873 s 2,282 s 2,232 s 2,702 s
2,357 s 1,701 s 1,814 s 1,767 s 1,891 s 1,914 s 3,048 s 3,033 s 2,936 s 2,829 s
2,282 s 1,568 s 1,500 s 1,538 s 1,119 s 2,062 s 2,185 s 2,133 s 2,473 s 2,967 s
1,024 s 2,515 s

Supplementary Fig. 11: Snapshots of simulations to examine ring formation probability with and without attraction of filaments to confining surface. Snapshots show maximum intensity projections at long enough times such that the bundle/loop configurations reach a stationary shape. All cases show concentration $c = 2\ \mu$M, cross-linking strength $k_{atr} = 2$ pN/μm with varying confining radius R, final filament length L, and persistence length l_P. **a** Simulations in small confinement ($R = 2.5\ \mu$m). We classified cases with circular arcs covering 75% of the circumference as ring-like. **b** Simulations in larger confinement ($R = 5\ \mu$m). Ring and ring-like configurations indicated by "R" and "RL". **c** Simulations as in panel B but with a higher individual filament persistence length ($l_p = 40\ \mu$m) to mimic the stiffer fascin bundles. Simulations with radii 2.5 and 5 μm began with 177 and 284 nuclei respectively.

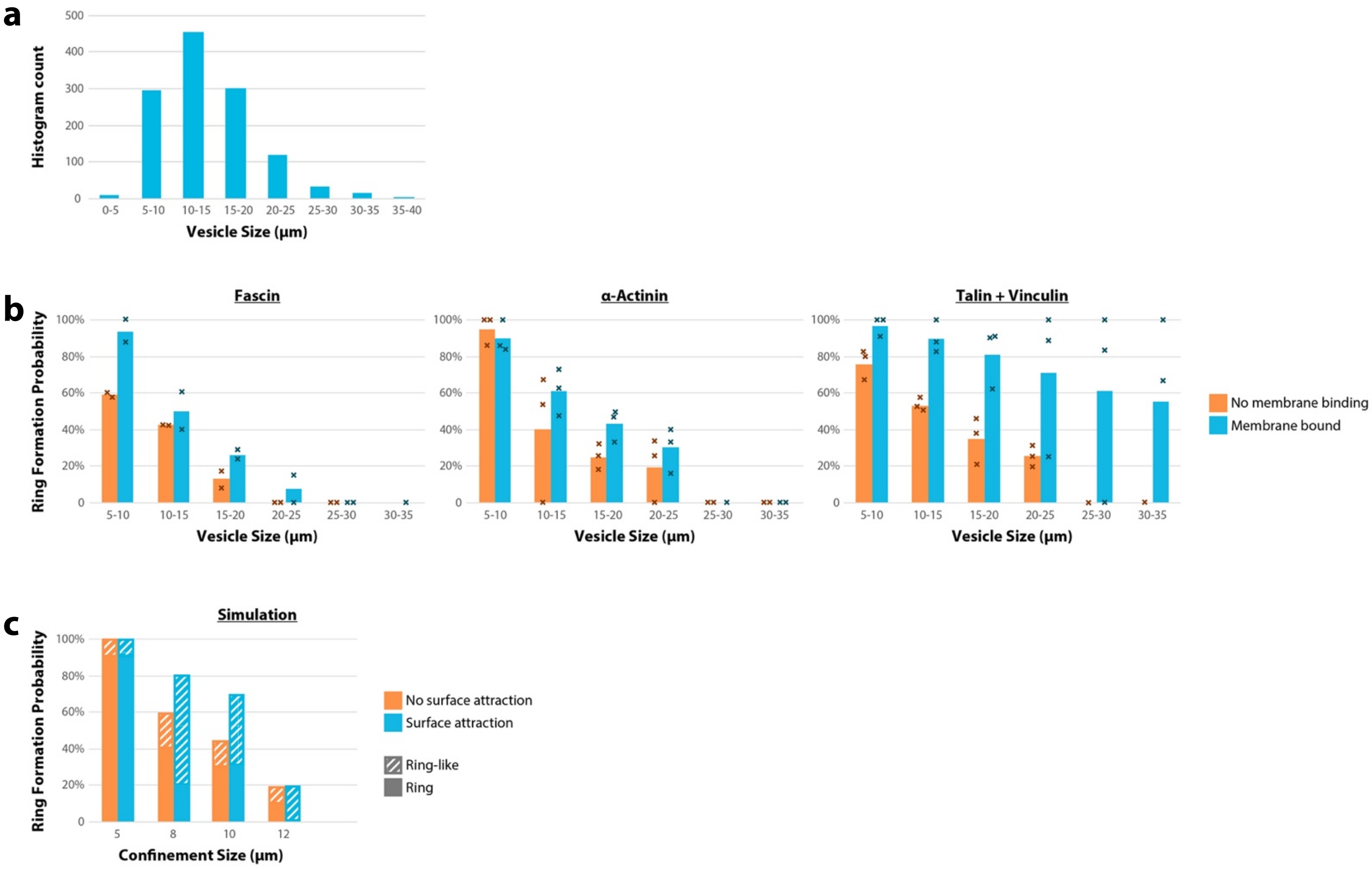

Supplementary Fig. 12: Dependency of ring formation probability on vesicle size. **a** Histogram showing the distribution of vesicle sizes (vesicle diameters). **b** Average probability of formation of single actin bundle rings in dependency of vesicle size. **c** Confinement size dependency of computer simulations. The x axis is diameter ($2R$). Simulations show concentration $c = 2\ \mu$M, cross-linking strength $k_{\text{atr}} = 2$ pN/μm with final filament length $L = 6$ µm, and persistence length $l_{\text{P}} = 17$ µm. For confinement sizes with diameters of 5, 8, and 10 µm, n = 20 for each condition (surface attraction and no surface attraction) and with a diameter of 12 µm n = 10 for each condition. The number of starting filament nuclei were 36, 145, 284 and 491 respectively.

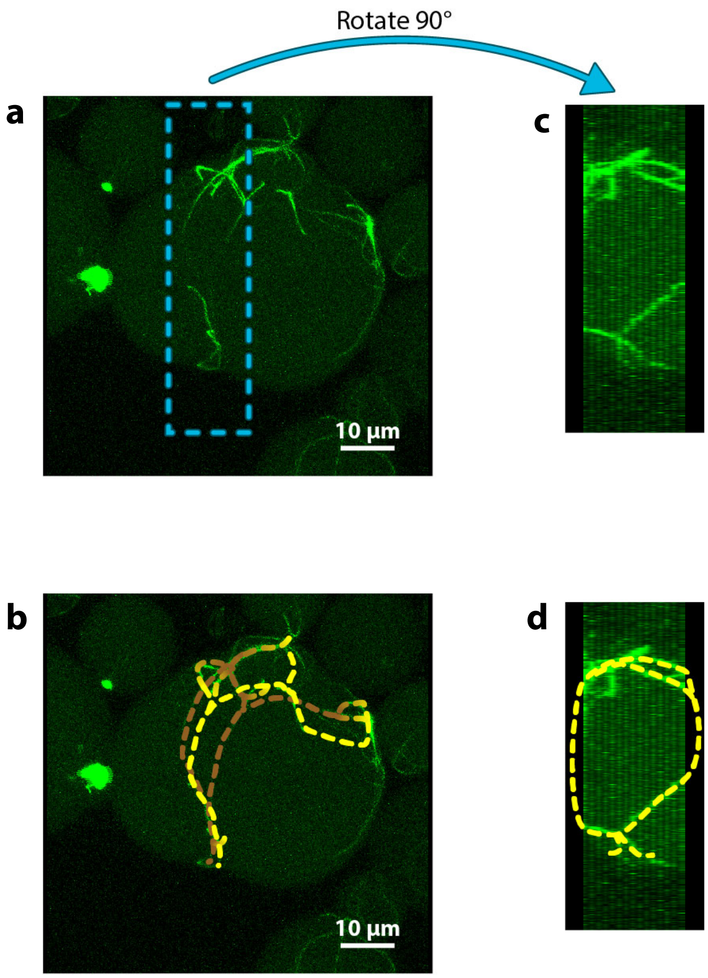

Supplementary Fig. 13: Microscopy representations illustrating the ring-like character of the actin bundles shown in Fig. 5a (at time point t = 60 min). **a** Projection of z-stack slices. Unfortunately we did not record the full volume (i.e. full z-range) of the vesicle, bottom and top are missing. **b** Overlay of suspected actin bundle network. Yellow: bundles in the upper half of the vesicle; brown: bundles towards bottom of vesicle (towards microcopy glass slide). **c** "side view" of center section (blue dotted rectangle in (**a**)). Z-stack was cropped to center area shown with blue dotted rectangle in (**a**). After 3D reconstruction was generated, viewing angle was determined to show a 90 degree angle compared to (**a**). **d** same image as (**c**), but again with yellow dotted lines indicating the suspected position of actin bundles, forming a ring within the vesicle.

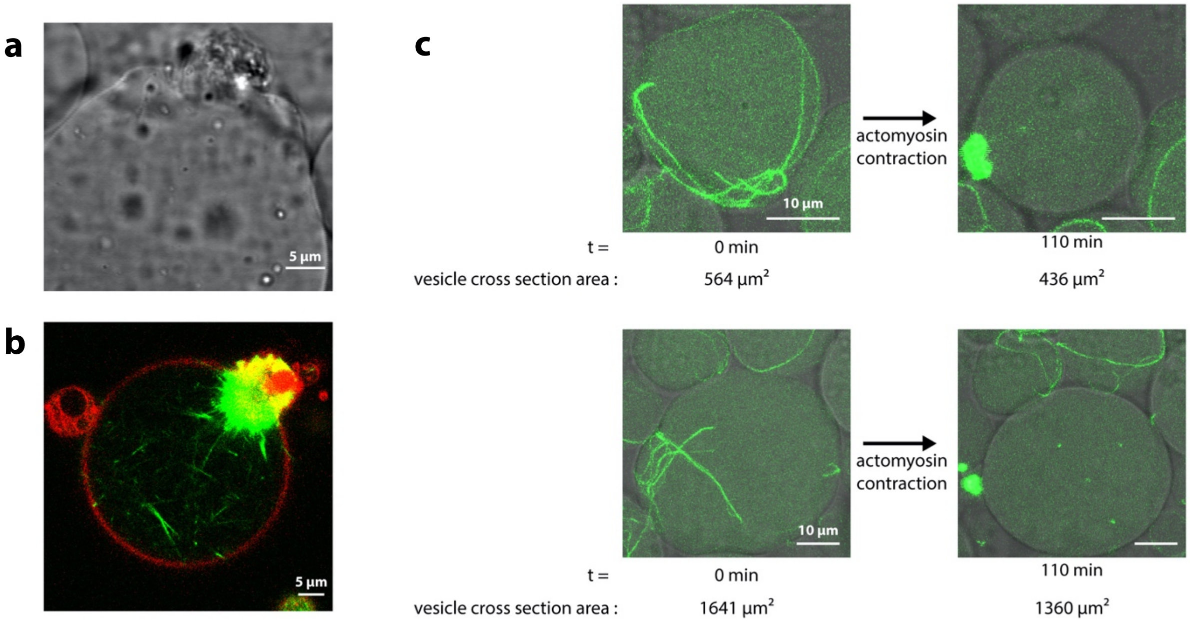

Supplementary Fig. 14: Membrane crumpling through actomyosin contraction. **a** Close-up of DIC image of the vesicle in Fig. 5a. Actomyosin deforms membrane and "crumples" it into one spot, thus presumably increasing membrane tension. **b** Experiment with actin bundled by fascin and contraction through myosin activity. Membrane (DOPE-ATTO655) in red. The vesicle's membrane concentrated into one spot in the upper right corner. **c** Two more examples of contracting actomyosin networks that cause a decrease in xy-cross section area through increasing membrane tension. We assume the vesicles become more spherical, thus becoming smaller in x and y, but taller in z-direction. While we did not record full z-stacks which allow to measure the full height of the vesicles, the phase contrast indicates that the vesicle is becoming taller: in the frames on the left (t = 0), the DIC image shows only a faint contour of the vesicle, indicating that the focal plane is approximately at the equator of the vesicle (the center of the vesicle is in focus), after contraction (t = 110 min) the vesicle contour resembles an outer shadow (negatively defocused), indicating that the equator of the vesicle shifted in +z direction.

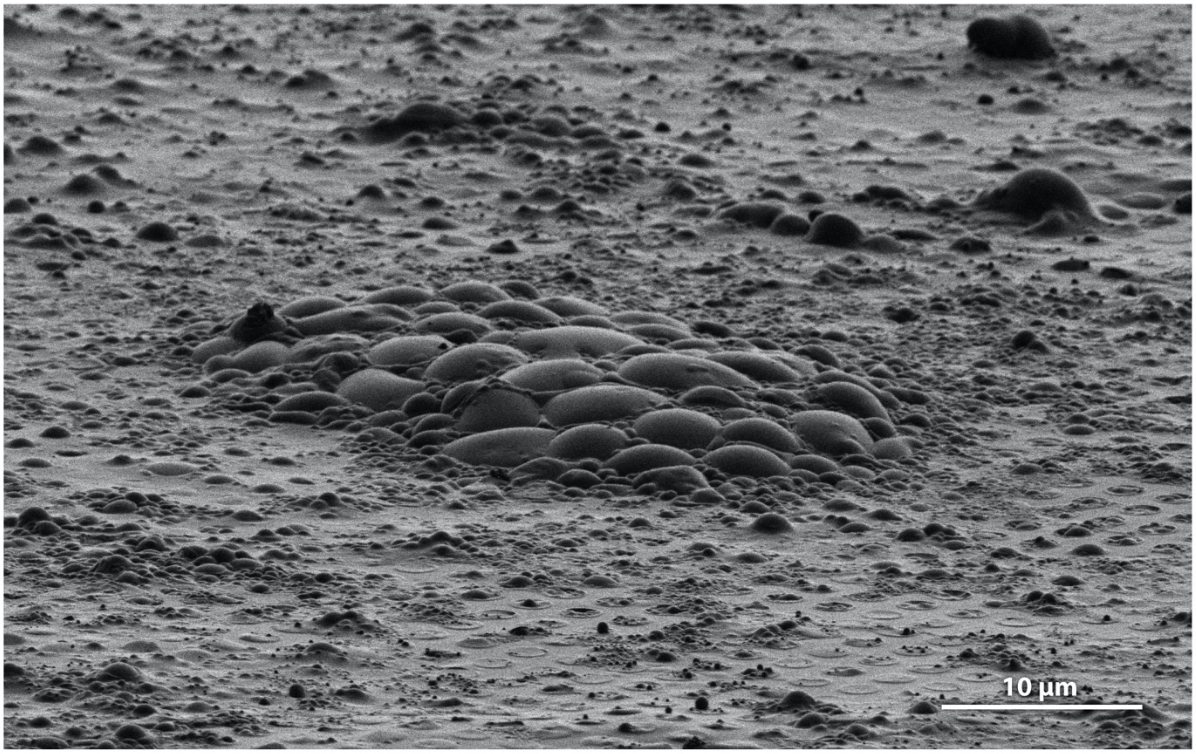

Supplementary Fig. 15: Rare example of vesicles that endured drying and freezing that do not contain an actin cortex. These visibly flattened vesicles contain short actin bundles that are not bound to the membrane (see Supplementary Fig. 6, 6 μM actin, 6.7% fascin, no membrane-binding).

Supplementary References

1 Adeli Koudehi, M., Rutkowski, D. M. & Vavylonis, D. Organization of Associating or Crosslinked Actin Filaments in Confinement. *Cytoskeleton* **76**, 532-548, doi:10.1002/cm.21565 (2019).

2 Schneider, C. A., Rasband, W. S. & Eliceiri, K. W. NIH Image to ImageJ: 25 years of image analysis. *Nature Methods* **9**, 671-675, doi:10.1038/nmeth.2089 (2012).

3 Schindelin, J. *et al.* Fiji: an open-source platform for biological-image analysis. *Nature Methods* **9**, 676-682, doi:10.1038/nmeth.2019 (2012).

4 Li, K. The image stabilizer plugin for ImageJ. *http://www.cs.cmu.edu/~kangli/code/Image_Stabilizer.html* (2008).

5 Rizk, A. *et al.* Segmentation and quantification of subcellular structures in fluorescence microscopy images using Squassh. *Nature Protocols* **9**, 586-596, doi:10.1038/nprot.2014.037 (2014).

6 Xu, T., Vavylonis, D. & Huang, X. 3D actin network centerline extraction with multiple active contours. *Medical Image Analysis* **18**, 272-284, doi:https://doi.org/10.1016/j.media.2013.10.015 (2014).

7 Xu, T. *et al.* SOAX: A software for quantification of 3D biopolymer networks. *Scientific Reports* **5**, 9081, doi:10.1038/srep09081 (2015).

8 Pettersen, E. F. *et al.* UCSF Chimera—A visualization system for exploratory research and analysis. *Journal of Computational Chemistry* **25**, 1605-1612, doi:10.1002/jcc.20084 (2004).
