# Supplementary material for: Reconstitution of contractile actomyosin rings in vesicles
Source: Nat Commun. 2021 Apr 15;12:2254. doi: 10.1038/s41467-021-22422-7 (PMC8050101; doi:10.1038/s41467-021-22422-7)
Supplement: Supplementary file 3 — Description of Additional Supplementary Files [file 41467_2021_22422_MOESM3_ESM.docx]

**Description of Additional Supplementary Files**

File Name: **Supplementary Movie 1**

Description: 3D pan video of image of many cytoskeletal vesicles (see Fig. 1b). Fascin-actin-bundles; not bound to the vesicle membrane. 2 µM actin, 0.3 µM fascin.

File Name: **Supplementary Movie 2**

Description: Montage of rotating views of both microscopy images and computer traced models of vesicles containing actin and various actin bundling proteins (see Fig. 1c). Bundles are not bound to the membrane. 2 µM actin; respectively 0.3 μM fascin, 0.9 μM VASP, 1 μM α-actinin, 2 μM talin and 2 μM vinculin.

File Name: **Supplementary Movie 3**

Description: Montage of rotating views of cytoskeletal vesicles with varying actin concentration (2 μM and 6 μM), fascin to actin ratios (3.3:100 to 15:100) and with and without actin-membrane binding (see Fig. 3a).

File Name: **Supplementary Movie 4**

Description: 3D pan video of image of many cytoskeletal vesicles (see Fig. 4c). Actin bundled by talin and vinculin; bundles are bound to the vesicle membrane. 2 µM actin, 2 µM talin, 2 µM vinculin.

File Name: **Supplementary Movie 5**

Description: Example of simulation with and without surface attraction for snapshots shown in Supplementary Fig. 7c (*c* = 2 µM, *L* = 6 µm, *R* = 5 µm, *l*_p_ = 40 µm and *k*_atr_ = 2 pN/ µm).

File Name: **Supplementary Movie 6**

Description: Contraction of actomyosin ring-like structure leads to furrow formation followed by contraction into dense cluster (see Fig. 5a). 2 µM actin, 2 µM talin, 2 µM vinculin, 0.1 µM myosin II.

File Name: **Supplementary Movie 7**

Description: Light-induced vesicle shape change through disassembly of actin bundles. Upon exposure to photo damage through increased laser power, cytoskeletal vesicles lose their stabilizing actin cortex and take on a round shape (see Fig. 6b).
