# Supplementary material for: Reconstitution of contractile actomyosin rings in vesicles
Source: Nat Commun. 2021 Apr 15;12:2254. doi: 10.1038/s41467-021-22422-7 (PMC8050101; doi:10.1038/s41467-021-22422-7)
Supplement: Supplementary file 11 — Reporting Summary [file 41467_2021_22422_MOESM11_ESM.pdf]

nature research

Corresponding author(s): Petra Schwille

Last updated by author(s): Feb 23, 2021

Reporting Summary

Nature Research wishes to improve the reproducibility of the work that we publish. This form provides structure for consistency and transparency in reporting. For further information on Nature Research policies, see our Editorial Policies and the Editorial Policy Checklist.

Statistics

For all statistical analyses, confirm that the following items are present in the figure legend, table legend, main text, or Methods section.

n/a	Confirmed	
☐	☒	The exact sample size (*n*) for each experimental group/condition, given as a discrete number and unit of measurement
☒	☐	A statement on whether measurements were taken from distinct samples or whether the same sample was measured repeatedly
☒	☐	The statistical test(s) used AND whether they are one- or two-sided<br>*Only common tests should be described solely by name; describe more complex techniques in the Methods section.*
☒	☐	A description of all covariates tested
☒	☐	A description of any assumptions or corrections, such as tests of normality and adjustment for multiple comparisons
☐	☒	A full description of the statistical parameters including central tendency (e.g. means) or other basic estimates (e.g. regression coefficient) AND variation (e.g. standard deviation) or associated estimates of uncertainty (e.g. confidence intervals)
☒	☐	For null hypothesis testing, the test statistic (e.g. *F*, *t*, *r*) with confidence intervals, effect sizes, degrees of freedom and *P* value noted<br>*Give P values as exact values whenever suitable.*
☒	☐	For Bayesian analysis, information on the choice of priors and Markov chain Monte Carlo settings
☒	☐	For hierarchical and complex designs, identification of the appropriate level for tests and full reporting of outcomes
☒	☐	Estimates of effect sizes (e.g. Cohen's *d*, Pearson's *r*), indicating how they were calculated

Our web collection on statistics for biologists contains articles on many of the points above.

Software and code

Policy information about availability of computer code

Data collection	Carl Zeiss - Zen 2.1 (black)
Data analysis	ImageJ 1.52u (Fiji)<br>Scientific Volume Imaging - Huygens Essential 19.10<br>UCSF Chimera<br>SOAX<br>MathWorks - MATLAB R2016a<br>Custom code is not central to the paper, but is made available with the paper.

For manuscripts utilizing custom algorithms or software that are central to the research but not yet described in published literature, software must be made available to editors and reviewers. We strongly encourage code deposition in a community repository (e.g. GitHub). See the Nature Research guidelines for submitting code & software for further information.

Data

Policy information about availability of data

All manuscripts must include a data availability statement. This statement should provide the following information, where applicable:

- Accession codes, unique identifiers, or web links for publicly available datasets
- A list of figures that have associated raw data
- A description of any restrictions on data availability

The data that support the findings of this study are available from the corresponding author upon reasonable request. An Excel file with the source data is provided with this paper.

Field-specific reporting

Please select the one below that is the best fit for your research. If you are not sure, read the appropriate sections before making your selection.

- [x] Life sciences
- [ ] Behavioural & social sciences
- [ ] Ecological, evolutionary & environmental sciences

For a reference copy of the document with all sections, see nature.com/documents/nr-reporting-summary-flat.pdf

Life sciences study design

All studies must disclose on these points even when the disclosure is negative.

Sample size	The ideal sample size could not be statistically determined prior to the experiments. Thus, sample sizes were chosen sufficiently large to draw meaningful conclusions. For analyses with high time and computational demands, a sample size of 3 to 5 was chosen, allowing for a reasonable statistical significance. Often the parameter sweep of conditions within a set of similar experiments yielded similar results, further supporting that sample sizes were large enough.
Data exclusions	GUVs that lacked any fluorescently labeled content were excluded from analysis. GUVs that were visibly deformed from a spherical shape were excluded from analysis.
Replication	The qualitative results shown in microscopy images were replicated to confirm reproducibility. Mostly, the features shown were not reproduced with identical protein concentrations, but with similar conditions and in sum reflect on robust underlying mechanisms. Generally for vesicles shown in images, it should be assumed that only one independent experiment was performed with the exact condition shown (exact concentrations). In total, we performed >=30 independent experiments with fascin, >=15 independent experiments with α-actinin, >=10 independent experiments with talin + vinculin and 3 independent experiments with VASP that all showed similar actin morphologies, as evidenced by the respective images. All images in Figure 1 were reproduced at least three times with similar concentrations. Results in Figure 2 were obtained on four different vesicles for each condition within two experimental runs. Conditions as in Figure 3 were varied once with a total of 20 conditions and incremental differences between the parameters, indicates a high degree of reproducibility. Experiments in Figure 4 were performed 2 (fascin) or 3 (α-actinin, talin + vinculin) times. Reproducibility of experiments with myosin was poor, as described in the paper. Figure 5a shows the most “furrow-like” membrane deformation we observed. We captured time series of membrane deformation in 4 additional cases. Membrane deformations as shown in Figure 6a were observed on many occasions in >=5 experimental runs. Result shown in Figure 6b was repeated many times within >=3 experimental runs. Cryo-EM shown in 6c was repeated once with similar results. Experiments shown in Supplementary Figures 1 to 4 were performed once. Images in Supplementary Figure 6 are from three different experimental runs per condition. Images shown in Supplementary Figures 8 to 10 were reproduced at least once with similar concentrations. Images in Supplementary Figures 13 to 15 were not reproduced.
Randomization	Samples were mixed from scratch. No allocation is done, randomization not applicable due to the type of experiments performed and the nature of the study.
Blinding	Investigators were not blinded, but strict parameters were defined for characterization of actin morphologies. For example in Figure 4a, actin rings were strictly defined as described in the paper. These strict definitions were used to safeguard against any implicit bias during data analysis.

Reporting for specific materials, systems and methods

We require information from authors about some types of materials, experimental systems and methods used in many studies. Here, indicate whether each material, system or method listed is relevant to your study. If you are not sure if a list item applies to your research, read the appropriate section before selecting a response.

Materials & experimental systems

n/a	Involved in the study	
[x]	[ ]	Antibodies
[x]	[ ]	Eukaryotic cell lines
[x]	[ ]	Palaeontology and archaeology
[x]	[ ]	Animals and other organisms
[x]	[ ]	Human research participants
[x]	[ ]	Clinical data
[x]	[ ]	Dual use research of concern

Methods

n/a	Involved in the study	
[x]	[ ]	ChIP-seq
[x]	[ ]	Flow cytometry
[x]	[ ]	MRI-based neuroimaging
